# Effectiveness of ChatGPT-4o in developing continuing professional development plans for graduate radiographers: a descriptive study

**DOI:** 10.3352/jeehp.2024.21.34

**Published:** 2024-11-18

**Authors:** Minh Chau, Elio Stefan Arruzza, Kelly Spuur

**Affiliations:** 1 Faculty of Science and Health, Charles Sturt University, Bathurst, NSW, Australia; 2 UniSA Allied Health & Human Performance, University of South Australia, Adelaide, SA, Australia; Hallym University, Korea

**Keywords:** Artificial intelligence, Cognition, Professional education, Radiography, X-ray computed tomography

## Abstract

**Purpose:**

This study evaluates the use of ChatGPT-4o in creating tailored continuing professional development (CPD) plans for radiography students, addressing the challenge of aligning CPD with Medical Radiation Practice Board of Australia (MRPBA) requirements. We hypothesized that ChatGPT-4o could support students in CPD planning while meeting regulatory standards.

**Methods:**

A descriptive, experimental design was used to generate 3 unique CPD plans using ChatGPT-4o, each tailored to hypothetical graduate radiographers in varied clinical settings. Each plan followed MRPBA guidelines, focusing on computed tomography specialization by the second year. Three MRPBA-registered academics assessed the plans using criteria of appropriateness, timeliness, relevance, reflection, and completeness from October 2024 to November 2024. Ratings underwent analysis using the Friedman test and intraclass correlation coefficient (ICC) to measure consistency among evaluators.

**Results:**

ChatGPT-4o generated CPD plans generally adhered to regulatory standards across scenarios. The Friedman test indicated no significant differences among raters (P=0.420, 0.761, and 0.807 for each scenario), suggesting consistent scores within scenarios. However, ICC values were low (–0.96, 0.41, and 0.058 for scenarios 1, 2, and 3), revealing variability among raters, particularly in timeliness and completeness criteria, suggesting limitations in the ChatGPT-4o’s ability to address individualized and context-specific needs.

**Conclusion:**

ChatGPT-4o demonstrates the potential to ease the cognitive demands of CPD planning, offering structured support in CPD development. However, human oversight remains essential to ensure plans are contextually relevant and deeply reflective. Future research should focus on enhancing artificial intelligence’s personalization for CPD evaluation, highlighting ChatGPT-4o’s potential and limitations as a tool in professional education.

## Graphical abstract

[Fig f2-jeehp-21-34]

## Introduction

### Background/rationale

Continuing professional development (CPD) is a core requirement for radiographers in Australia, regulated by the Medical Radiation Practice Board of Australia (MRPBA). CPD ensures that practitioners maintain and enhance their skills, knowledge, and professional competence throughout their careers [1]. According to the MRPBA guidelines, radiographers must complete 60 hours of CPD over 3 years, with at least 10 hours documented per year [1]. Additionally, CPD plans must be reflective, varied, and aligned with the individual’s scope of practice to ensure relevance and patient care quality. For final-year radiography students, preparing a CPD plan is essential to transitioning to professional practice, helping them build a roadmap for their professional development during their graduate year. This requirement informs program accreditation to demonstrate that students have thoughtfully developed a CPD plan, preparing them to operate as independent, autonomous, and competent practitioners [2].

However, many students find it challenging to create a structured, reflective, and compliant CPD plan that aligns with professional expectations and their career goals [3,4]. Issues such as balancing multiple commitments, managing their time effectively, and understanding the nuances of reflective practice complicate the process [3]. These challenges are further compounded by the need to ensure that their plans meet the specific requirements outlined by the MRPBA, making CPD planning a potentially daunting task for novice practitioners. In this context, generative artificial intelligence (AI) tools such as ChatGPT-4o present a promising opportunity to assist students in formulating effective CPD plans. This innovation has recently been trialed in teaching, science, and information technology [5]. ChatGPT-4o can provide personalized suggestions, facilitate reflective writing, and offer guidance on structuring and documenting CPD activities. By generating customized prompts, summarizing CPD requirements, and assisting with reflective documentation, ChatGPT-4o can empower students to develop plans that are not only aligned with regulatory expectations but are also tailored to their personal and professional needs.

### Objectives

This research explores the potential of ChatGPT-4o to support diagnostic radiography students in the development of CPD plans for their graduate year. It examines how ChatGPT-4o can simplify planning, enhance student engagement with reflective practices, and provide targeted recommendations for CPD activities. By leveraging AI, students may overcome common challenges associated with CPD planning and gain confidence in their ability to manage lifelong professional development. In doing so, this research aims to contribute to the growing conversation on the role of AI in medical education and its potential to streamline critical aspects of academic and professional preparation.

## Methods

### Ethics statement

This study does not involve human participants or personal data, so formal ethical approval was not required. However, the use of ChatGPT-4o outputs is accompanied by a disclaimer that AI-generated plans should not replace critical judgment and must be evaluated for practical use.

### Study design

This descriptive study focuses on using ChatGPT-4o to generate 3 tailored 3-year CPD plans that align with the MRPBA CPD guidelines for radiographers. The CPD plans address the specific requirements, challenges, and development goals of 3 hypothetical graduate radiographers. A panel of academics evaluated each plan to determine its quality, alignment with professional requirements, and suitability for practical implementation.

### Setting

The study adopts a quantitative design to assess how effectively ChatGPT-4o can generate structured, appropriate, and timely CPD plans. Three different graduate radiographer scenarios were created, each with unique practice environments and career goals. ChatGPT-4o generated a tailored CPD plan for each scenario, covering 3 years, including specialization in computed tomography (CT) in the second yearfrom October 2024 to November 2024.

### Participants

Raters were 3 co-authors of this study. They registered with MRPBA, with expertise in medical radiation science.

### Variables

The primary outcome was the rating scale by raters on the ChatGPT-4o’s answers.

### Data sources/measurements

#### Scenarios for CPD plans

Three distinct CPD plans were generated using ChatGPT-4o to align with MRPBA guidelines for graduate radiographers in diverse healthcare settings (Supplement 1). Each plan includes annual activities focusing on CT specialization by the second year to meet MRPBA’s 60-hour CPD requirement over 3 years [1].

In Scenario 1, a radiographer in a private outpatient clinic focuses on patient throughput and communication, progressing to CT training and quality improvement projects by the third year. Scenario 2 involves a radiographer in a private hospital handling both inpatients and outpatients, with additional on-call duties. This plan includes basic life support, intravenous cannulation, CT training, and multi-modal imaging conferences. Scenario 3 features a radiographer in a public hospital working 24-hour shifts, emphasizing trauma imaging, fatigue management, and cultural competence training. These scenarios demonstrate ChatGPT-4o’s capacity to generate tailored CPD plans suited to various clinical contexts, incorporating hands-on training, reflection, and professional interactions and meeting MRPBA requirements (Tables 1–3).

#### Evaluation by an academic panel

A panel of 3 academics was recruited to review the CPD plans generated by ChatGPT-4o from October 2024 to November 2024. Each plan was evaluated using 5 critical criteria developed by the authors, aligned with professional requirements and practical application. Appropriateness assessed whether the plan aligned with the graduate radiographer’s specific workplace environment and career goals. Timeliness evaluated whether the CPD activities were logically spaced over the 3 years, reflecting a natural progression toward specialization in CT by the second year. Relevance focused on whether the activities matched the radiographer’s development needs and adhered to the MRPBA’s CPD requirements. Reflection determined whether the plan incorporated reflective practices aligned with MRPBA guidelines. Completeness ensured that the plan covered 60 hours of CPD over 3 years, with at least 10 hours completed annually.

Each panel rated the plans on a scale from 0 to 10 for each criterion, where 0 indicated non-compliance, and 10 represented exemplary alignment. The average score across the 5 criteria was calculated to determine the overall quality of each CPD plan (Table 4).

### Bias

The authors participated in the evaluation as raters. Therefore, there is a tendency to evaluate more positively.

### Study size

No sample size estimation was required since this was a descriptive study.

### Statistical methods

A descriptive analysis of the scores provided by the academic panel was performed. For the quantitative variables (scores for each CPD plan), the mean±standard deviation (SD) was reported, depending on whether the distribution was normal or non-normal. Data distribution was tested using the Kolmogorov-Smirnov test with Lilliefors correction, with a significance level of P<0.05. To assess the consistency of ratings between the 3 raters across each scenario, we used the Friedman test, a non-parametric test suitable for analyzing differences in ordinal or non-normally distributed data across related groups. The Friedman test was conducted separately for each scenario to evaluate whether there were statistically significant differences in scores assigned by the raters for each criterion. To assess inter-rater reliability among the 3 academic reviewers, we calculated the intraclass correlation coefficient (ICC) using a 2-way mixed with absolute agreement, assuming a 95% confidence interval (CI). The ICC results were interpreted according to the ranges established by Koo and Li [6] in 2016: <0.5, low reliability; 0.5 to 0.75, moderate agreement; 0.75 to 0.9, good agreement; >0.9, excellent reliability.

## Results

### Descriptive statistics

**Fig. 1.**
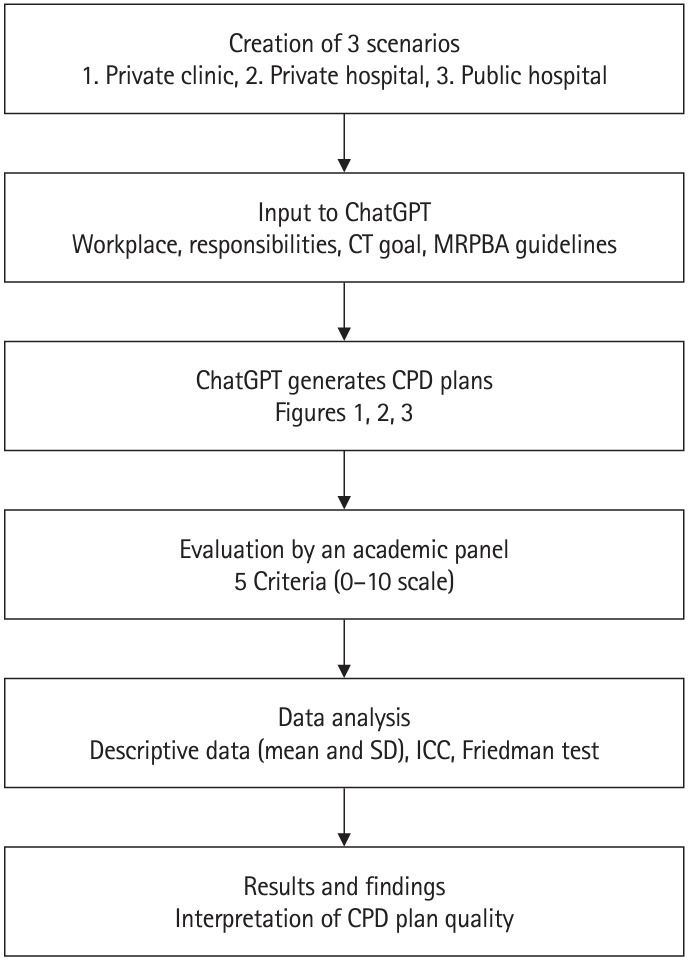
Flowchart of methodology for continuing professional development (CPD) plan generation and evaluation. CT, computed tomography; MRPBA, Medical Radiation Practice Board of Australia; SD, standard deviation; ICC, intraclass correlation coefficient.

Fig. 1 summarizes the methodology, outlining steps from creating 3 scenarios (a private clinic, private hospital, and public hospital) to generating and evaluating CPD plans with ChatGPT-4o. Three academics assessed these plans based on 5 criteria: appropriateness, timeliness, relevance, reflection, and completeness.

Table 5 presents scores from each rater for the 3 scenarios, including overall and individual criterion scores. Rater 1’s overall scores ranged from 34 to 40, rater 2 from 37 to 40, and rater 3 from 37 to 40. Average scores spanned from 6.8 (SD=2.8) to 8 (SD=1.3). While relevance and reflection showed consistent scores between 8 and 10, timeliness and completeness varied more, with timeliness ranging from 3 to 10 and completeness also showing fluctuations across raters and scenarios.

### Main results

We conducted a Friedman test and ICC analysis to evaluate the consistency of ratings between the 3 raters across each scenario. The Friedman test revealed no statistically significant differences in ratings among the raters for any of the scenarios: Scenario 1, χ^2^ (2)=1.733, P=0.420; Scenario 2, χ^2^ (2)=0.545, P=0.761; and Scenario 3, χ^2^ (2)=0.429, P=0.807. This result suggests that the scores provided by the raters were consistent within each scenario.

The ICC analysis, using a 2-way mixed-effects model for absolute agreement, indicated low levels of agreement across raters in all scenarios. For Scenario 1, the ICC for individual ratings was –0.19 (95% CI, –0.51 to 0.61), and the average rating ICC was –0.96 (95% CI, –111.36 to 0.82), with no significant agreement (F(4.0, 8.0)=0.56, P=0.697). In Scenario 2, the ICC for individual ratings was 0.19 (95% CI, –0.41 to 0.84), and the average rating ICC was 0.41 (95% CI, –6.51 to 0.94), also indicating no significant agreement (F(4.0, 8.0)=1.55, P=0.277). For Scenario 3, the ICC for individual ratings was 0.02 (95% CI, –0.49 to 0.78), and the average rating ICC was 0.06 (95% CI, –72.17 to 0.91), again showing no significant agreement (F(4.0, 8.0)=1.05, P=0.440). These results indicate low inter-rater reliability across all scenarios, highlighting variability in rater scoring.

## Discussion

### Key results

It is the first descriptive, experimental study exploring the use of generative AI, specifically ChatGPT-4o, to support radiography students in developing CPD plans tailored to their future roles. Given the challenges graduate radiographers face in creating CPD plans that align with the requirements of the MRPBA while also meeting their own professional goals, this study provides important insights into the potential benefits and limitations of using AI to streamline CPD planning [1]. ChatGPT-4o generated structured CPD plans that aligned with MRPBA guidelines across 3 distinct scenarios, simulating different radiography work environments. However, the results revealed variability in how academic raters evaluated these AI-generated plans, particularly in the timeliness and completeness criteria. Despite the Friedman test indicating no statistically significant differences in ratings among the 3 raters for any scenario, the low ICC values across all scenarios suggest that raters differed in their subjective interpretations of the plans’ quality.

### Interpretation

These findings highlight both the potential and limitations of ChatGPT-4o as a tool for CPD planning. The ICC findings indicate that while ChatGPT-4o-generated plans meet basic regulatory guidelines, they may lack the nuanced depth and context that human experts seek in high-quality CPD plans. For example, the low ICC values observed in this study for individual and average ratings (range, –0.19 to 0.41) suggest poor agreement among raters, particularly in assessing timeliness and completeness. This inconsistency may stem from ChatGPT-4o’s limitations in capturing the personalized and contextual aspects of CPD activities, especially when it comes to aligning activities with an individual’s specific learning trajectory and career development goals. This variation likely reflects the challenges associated with assessing CPD plans that contain reflective components—a known area of difficulty for students, as previous research has noted that students often lack the depth required for meaningful reflections [7]. The inconsistencies in ratings, especially regarding reflection and completeness, highlight the subjective nature of evaluating CPD tasks.

### Comparison with previous studies

Generative AI, huge language models like ChatGPT-4o, has been increasingly explored for its potential to enhance learning in higher education, particularly in diagnostic radiography [8]. ChatGPT-4o is a conversational agent that can generate human-like text based on prompts. It is a versatile tool for various educational tasks, including personalized tutoring, content generation, and academic writing support [9]. Studies have shown that AI tools can assist students by providing instant feedback, helping them organize thoughts, and even guiding them through complex academic tasks [10]. These findings align with observations by Karas et al. [3], who report that students often face difficulties in structuring CPD plans that are both comprehensive and strategically timed, particularly without explicit guidance. These findings are significant in fields such as medical radiation science, where practitioners are expected to stay current with rapidly advancing technologies and clinical practices.

### Limitations

This study’s primary limitation lies in the subjective evaluation process, as reflected in rater variability, which may introduce bias. Low ICC values point to inconsistent interpretations of criteria like timeliness and completeness, suggesting potential imprecision in assessment. ChatGPT-4o also has limitations in generating contextually rich reflections and personalized guidance, potentially affecting the quality of the plans. Furthermore, student obligations like part-time work and family responsibilities may hinder full engagement with the CPD process [11]. The 5 evaluation criteria were developed through consensus among 3 academic experts and should be further validated in future studies to ensure broader applicability and reliability.

### Generalizability

The findings are relevant to other educational contexts where CPD planning and reflection are crucial, particularly in healthcare. While specific to Australian diagnostic radiography students, ChatGPT-4o’s role in foundational CPD planning may benefit global health disciplines. In professional education, AI tools show promise in supporting lifelong learning and CPD by helping users identify resources, structure CPD, and engage in reflection [12]. Given the diverse regulatory landscapes and expectations within healthcare professions, educators and institutions must, however, incorporate human oversight and tailored guidance to ensure that AI-generated plans meet each region’s specific regulatory standards and professional goals. Integrating AI in CPD planning could not only ease the logistical and cognitive demands of planning but also improve accessibility to structured guidance in resource-limited settings, promoting global alignment with best practices in professional education and continuous development.

### Suggestions

Given the challenges students face in developing CPD plans and the capabilities of AI tools such as ChatGPT-4o, there is significant potential for integration. ChatGPT-4o can assist students by generating tailored suggestions for CPD activities based on their scope of practice, helping them meet the MRPBA’s requirements. While AI tools in education are still relatively new, there is growing evidence that these tools can significantly enhance learning outcomes by offering personalized and timely support [13]. For instance, AI-driven tools can improve students’ ability to plan and organize complex tasks by breaking them down into manageable steps [14]. However, there are ethical considerations surrounding the use of AI in education, particularly regarding over-reliance on AI-generated content. While ChatGPT-4o can assist students in generating ideas and structuring their CPD plans, it is essential to ensure that students remain actively engaged in the reflective process and avoid passive consumption of AI-generated materials [15]. Educators must guide students in critically assessing the suggestions provided by AI tools and encourage them to maintain ownership of their learning journey.

### Conclusion

In conclusion, this study provides a preliminary exploration into the role of generative AI, specifically ChatGPT-4o, in supporting radiography students with CPD planning aligned with MRPBA requirements. ChatGPT-4o demonstrated potential in generating structured CPD plans tailored to various clinical scenarios, offering foundational guidance on organizing CPD activities and encouraging reflective practices. However, variability in ratings and low ICC values indicate inconsistencies in raters’ interpretations of AI-generated plans. This inconsistency suggests that while ChatGPT-4o can reduce the cognitive load associated with CPD planning, human oversight remains critical to ensure contextual relevance, personalized depth, and meaningful engagement in reflective practice.

## Figures and Tables

**Figure f2-jeehp-21-34:**
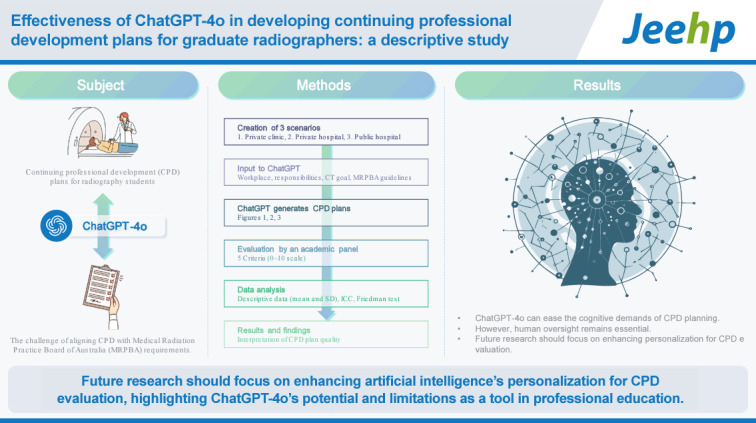


**Table 1.** Continuing professional development plan based on scenario 1: graduate radiographer in a private clinic (outpatient only)

Year	CPD activities	Type (substantive/general)	Time (hr)	Reflection component
Year 1	Attend introductory workshop on patient communication and workflow management	Substantive	5	Reflect on improving patient throughput
	Complete online course on radiation safety for outpatient settings	Substantive	5	Document key takeaways for future reference
	Join peer discussion group on outpatient radiography challenges	General	5	Write reflective notes post-discussion
Year 2	Enroll in a specialized course on CT	Substantive	10	Reflect on how CT knowledge integrates into daily practice
	Participate in hands-on CT applications training	Substantive	5	Evaluate learning outcomes and areas for improvement
	Present a case study on outpatient care at a local conference	Substantive	10	Analyze feedback from peers on the presentation
Year 3	Attend CT-specific conference (focused on outpatient cases)	Substantive	10	Record reflective insights in the CPD log
	Participate in quality improvement project in outpatient imaging	Substantive	10	Reflect on how changes impacted patient experience

CPD, continuing professional development; CT, computed tomography.

**Table 2.** Continuing professional development plan based on scenario 2: graduate radiographer in a private hospital (inpatient, outpatient, on-call services)

Year	CPD activities	Type (substantive/general)	Time (hr)	Reflection component
Year 1	Complete basic life support and intravenous cannulation course	Substantive	10	Reflect on new skills applied in clinical settings
	Participate in peer mentoring program for managing on-call duties	General	5	Document learning and time management strategies
	Join a webinar on patient care for inpatients and outpatients	General	5	Reflect on strategies for different patient groups
Year 2	Enroll in CT specialization program	Substantive	10	Document progress in learning CT protocols
	Participate in simulation training for CT in emergency cases	Substantive	5	Evaluate learning outcomes in the simulation log
	Present at in-service training on radiographic protocols	Substantive	10	Reflect on peer feedback from the presentation
Year 3	Attend CT conference focusing on multi-modal imaging	Substantive	10	Record reflections on new techniques learned
	Collaborate on an audit of radiology department practices	Substantive	5	Reflect on contributions to process improvements
	Complete training on patient interaction during on-call services	General	5	Write reflective notes on patient experiences

CPD, continuing professional development; CT, computed tomography.

**Table 3.** Continuing professional development plan based on scenario 3: graduate radiographer in a public hospital (24-hour shift work)

Year	CPD activities	Type (substantive/general)	Time (hr)	Reflection component
Year 1	Attend a workshop on managing fatigue and self-care for shift workers	General	5	Reflect on strategies to manage fatigue
	Complete training on trauma imaging techniques	Substantive	10	Reflect on improvements in emergency cases
	Join a peer-led journal club on radiography advancements	General	5	Write summaries of discussed research articles
Year 2	Enroll in an advanced CT course with a focus on emergency imaging	Substantive	10	Document reflections on emergency imaging skills
	Participate in simulation-based CT training for critical care cases	Substantive	5	Reflect on handling emergency scenarios
	Present at public hospital symposium on imaging best practices	Substantive	10	Evaluate presentation outcomes and feedback
Year 3	Attend a conference on CT innovations in public healthcare	Substantive	10	Record insights in CPD logbook
	Join a project team reviewing radiology protocols for shifts	Substantive	5	Reflect on contributions to project outcomes
	Complete training on cultural competence and patient care	General	5	Reflect on strategies for diverse patient care

CPD, continuing professional development; CT, computed tomography.

**Table 4.** Continuing professional development plan evaluation tool

Criterion	Evaluation question	Rating (0–10)
Appropriateness	Does the plan align with the radiographer’s workplace environment and career goals?	
Timeliness	Are the CPD activities logically spaced over the 3 years, with a clear progression toward CT?	
Relevance	Are the activities relevant to the radiographer’s professional development and aligned with MRPBA requirements?	
Reflection	Does the plan incorporate reflective practices that meet the MRPBA’s guidelines for meaningful reflection?	
Completeness	Does the plan cover the required 60 CPD hours over 3 years, with at least 10 hours annually?	

CPD, continuing professional development; CT, computed tomography; MRPBA, Medical Radiation Practice Board of Australia.

**Table 5.** Summary of scores provided by the academic panel

	Rater 1			Rater 2			Rater 2		
	Scenario 1	Scenario 2	Scenario 3	Scenario 1	Scenario 2	Scenario 3	Scenario 1	Scenario 2	Scenario 3
Overall score	34	37	38	40	37	38	40	37	37
Average±SD score	6.8±2.8	7.4±1.8	7.6±2.1	8±2.1	7.4±3	7.6±2.6	8±1	7.4±1.3	7.4±1.1
Appropriateness	5	6	6	7	6	6	7	6	7
Timeliness	3	5	5	10	10	10	9	8	7
Relevance	8	8	8	8	8	8	8	8	8
Reflection	8	9	9	10	10	10	9	9	9
Completeness	10	9	10	5	3	4	7	6	6

SD, standard deviation.

## Supplementary materials

Supplementary files are available from Harvard Dataverse: https://doi.org/10.7910/DVN/BRQFXF

Supplement 1. Prompts and responses for a continuing professional development plan generation.

Supplement 2. Audio recording of the abstract.
