# Supplementary material for: Effectiveness of ChatGPT-4o in developing continuing professional development plans for graduate radiographers: a descriptive study
Source: J Educ Eval Health Prof. 2024 Nov 18;21:34. doi: 10.3352/jeehp.2024.21.34 (PMC11637979; doi:10.3352/jeehp.2024.21.34)
Supplement: Supplementary file 1 — Supplement 1. Prompts and responses for a continuing professional development plan generation. [file jeehp-21-34-suppl1.pdf]

Supplement 1. Prompts and responses for CPD plan generation

Prompt 1: Scenario 1 - Graduate Radiographer in Private Clinic (Outpatient Only)

Prompt: "I am developing a continuing professional development (CPD) plan for a graduate radiographer who will be working in a private clinic that only deals with outpatient settings. The CPD plan should span over 3 years, with a focus on meeting the MRPBA guideline of 60 hours over 3 years (10 hours minimum per year). Include opportunities for reflective practice. The radiographer aims to specialize in computed tomography (CT) by the second year. Please generate a CPD plan that aligns with these requirements. See attached CPD guideline for MRPBA for further information."

Response: Year 1:

- **Activity 1**: Attend introductory workshop on patient communication and workflow management (Substantive, 5 hours).
    - *Reflection*: Reflect on improving patient throughput.
- **Activity 2**: Complete an online course on radiation safety for outpatient settings (Substantive, 5 hours).
    - *Reflection*: Document key takeaways for future reference.
- **Activity 3**: Join peer discussion group on outpatient radiography challenges (General, 5 hours).
    - *Reflection*: Write reflective notes post-discussion.

Year 2:

- **Activity 1**: Enroll in a specialized course on computed tomography (CT) (Substantive, 10 hours).
    - *Reflection*: Reflect on how CT knowledge integrates into daily practice.
- **Activity 2**: Participate in hands-on CT applications training (Substantive, 5 hours).
    - *Reflection*: Evaluate learning outcomes and areas for improvement.
- **Activity 3**: Present a case study on outpatient care at a local conference (Substantive, 10 hours).
    - *Reflection*: Analyze feedback from peers on presentation.

Year 3:

- **Activity 1**: Attend CT-specific conference (focused on outpatient cases) (Substantive, 10 hours).
    - *Reflection*: Record reflective insights in CPD log.

- **Activity 2**: Participate in a quality improvement project in outpatient imaging (Substantive, 10 hours).
    - *Reflection*: Reflect on how changes impacted patient experience.

Prompt 2: Scenario 2 - Graduate Radiographer in Private Hospital (Inpatient, Outpatient, On-Call Services)

Prompt: "I am developing a CPD plan for a graduate radiographer who will be working in a private hospital. The hospital serves both inpatients and outpatients, and the radiographer will also be responsible for after-hours on-call duties. The CPD plan should span over 3 years, with a focus on meeting the MRPBA guideline of 60 hours over 3 years (10 hours minimum per year). Include opportunities for reflective practice. The radiographer aims to specialize in computed tomography (CT) by the second year. Please generate a CPD plan that aligns with these requirements. See attached CPD guideline for MRPBA for further information."

Response: Year 1:

- **Activity 1**: Complete Basic Life Support (BLS) and IV Cannulation course (Substantive, 10 hours).
    - *Reflection*: Reflect on new skills applied in clinical settings.
- **Activity 2**: Participate in a peer mentoring program for managing on-call duties (General, 5 hours).
    - *Reflection*: Document learning and time management strategies.
- **Activity 3**: Join a webinar on patient care for inpatients and outpatients (General, 5 hours).
    - *Reflection*: Reflect on strategies for different patient groups.

Year 2:

- **Activity 1**: Enroll in a computed tomography (CT) specialization program (Substantive, 10 hours).
    - *Reflection*: Document progress in learning CT protocols.
- **Activity 2**: Participate in simulation training for CT in emergency cases (Substantive, 5 hours).
    - *Reflection*: Evaluate learning outcomes in simulation log.
- **Activity 3**: Present at in-service training on radiographic protocols (Substantive, 10 hours).

- *Reflection*: Reflect on peer feedback from presentation.

Year 3:

- **Activity 1**: Attend a CT conference focusing on multi-modal imaging (Substantive, 10 hours).
  - *Reflection*: Record reflections on new techniques learned.
- **Activity 2**: Collaborate on an audit of radiology department practices (Substantive, 5 hours).
  - *Reflection*: Reflect on contributions to process improvements.
- **Activity 3**: Complete training on patient interaction during on-call services (General, 5 hours).
  - *Reflection*: Write reflective notes on patient experiences.

Prompt 3: Scenario 3 - Graduate Radiographer in Public Hospital (24-Hour Shift Work)

Prompt: "I am creating a CPD plan for a graduate radiographer who will be working in a public hospital with a 24-hour shift roster. The CPD plan should span over 3 years, with a focus on meeting the MRPBA guideline of 60 hours over 3 years (10 hours minimum per year). Include opportunities for reflective practice. The radiographer aims to specialize in computed tomography (CT) by the second year. Please generate a CPD plan that aligns with these requirements. See attached CPD guideline for MRPBA for further information."

Response: Year 1:

- **Activity 1**: Attend a workshop on managing fatigue and self-care for shift workers (General, 5 hours).
  - *Reflection*: Reflect on strategies to manage fatigue.
- **Activity 2**: Complete training on trauma imaging techniques (Substantive, 10 hours).
  - *Reflection*: Reflect on improvements in emergency cases.
- **Activity 3**: Join a peer-led journal club on radiography advancements (General, 5 hours).
  - *Reflection*: Write summaries of discussed research articles.

Year 2:

- **Activity 1**: Enroll in an advanced CT course with a focus on emergency imaging (Substantive, 10 hours).
  - *Reflection*: Document reflections on emergency imaging skills.

- **Activity 2**: Participate in simulation-based CT training for critical care cases (Substantive, 5 hours).
    - *Reflection*: Reflect on handling emergency scenarios.
- **Activity 3**: Present at a public hospital symposium on imaging best practices (Substantive, 10 hours).
    - *Reflection*: Evaluate presentation outcomes and feedback.

Year 3:

- **Activity 1**: Attend a conference on CT innovations in public healthcare (Substantive, 10 hours).
    - *Reflection*: Record insights in CPD logbook.
- **Activity 2**: Join a project team reviewing radiology protocols for shifts (Substantive, 5 hours).
    - *Reflection*: Reflect on contributions to project outcomes.
- **Activity 3**: Complete training on cultural competence and patient care (General, 5 hours).
    - *Reflection*: Reflect on strategies for diverse patient care.

Summary table. Input Prompts Used for Generating CPD Plans Using ChatGPT-4o

**Scenario**	**Workplace**	**Responsibilities and Key Focus**	**Professional Goal**	**MRPBA CPD Requirements Included**
**Scenario 1**	Private clinic (outpatients only)	Patient throughput, no on-call duties	Specialise in CT by Year 2	60 hours of CPD over 3 years, 10 hours minimum per year, reflective practice required
**Scenario 2**	Private hospital (inpatient/outpatient, on-call services)	Manage on-call duties and mixed patient care	Specialise in CT by Year 2	
**Scenario 3**	Public hospital (24-hour shift roster)	Manage shift work, exposure to emergency cases	Specialise in CT by Year 2	
